# Flexible Mechanical Sensors for Plant Growth Monitoring: An Emerging Area for Smart Agriculture

**DOI:** 10.3390/s24247995

**Published:** 2024-12-14

**Authors:** Thi Thu Hien Phan, Thi Mai Vi Ngo, Hoang-Phuong Phan

**Affiliations:** 1 School of Agriculture and Natural Resources, Vinh University, Vinh 43108, Vietnam; ptthien@vinhuni.edu.vn (T.T.H.P.);; 2 School of Mechanical and Manufacturing Engineering, The University of New South Wales, Sydney, NSW 2052, Australia; 3 Tyree Foundation Institute of Health Engineering, The University of New South Wales, Sydney, NSW 2052, Australia

**Keywords:** flexible electronics, smart agriculture, mechanical sensors, wireless communication, plant growth monitoring

## Abstract

The last decade has seen significant progress in the development of flexible electronics and sensors, particularly for display technologies and healthcare applications. Advancements in scalable manufacturing, miniaturization, and integration have further extended the use of this new class of devices to smart agriculture, where multimodal sensors can be seamlessly attached to plants for continuous and remote monitoring. Among the various types of sensing devices for agriculture, flexible mechanical sensors have emerged as promising candidates for monitoring vital parameters, including growth rates and water flow, providing a new avenue for understanding plant health and growth under varied environmental conditions. This perspective provides a snapshot of recent progress in this exciting and unconventional area of research and highlights potential opportunities for the future.

## 1. Introduction

The development of flexible electronics and sensors has seen rapid progress over the last decade, with broad applications spanning various areas, from bendable displays to wearable smart patches and implanted neuron stimulators and recorders [1,2]. Their unique capabilities for stretching and bending bring unprecedented experiences to users, both when utilized in virtual reality (VR) components and in creating better interfaces with objects of interest (e.g., human skin) in biomedical devices [3,4]. Representative examples of flexible electronics include wearable sweat sensors for on-site sweat analysis, acoustic sensors for body sound monitoring, and anemometers for shunt flow monitoring. These devices offer real-time, continuous monitoring, enabling the timely detection of abnormalities and diagnosis of potential diseases. One of the primary approaches to achieving mechanical flexibility involves the use of industrially standard semiconductors (such as silicon, silicon carbide, and gallium nitride) and metals (Au, Pt, Al) in the form of nanomembranes, which exhibit low bending stiffness and can be transferred onto soft polymers via mechanical stamping [5,6,7,8]. Another common method employs intrinsically stretchable conductive polymers (e.g., PEDOT-PSS and organic materials) for the development of soft functional components [9,10]. These engineering strategies, combined with advancements in signal processing and wireless communication, have driven the commercialization of flexible electronics, with the market size expected to reach USD 74 billion by 2030 [11]. 

These advancements in flexible electronics have expanded their applications to other fields, including smart agriculture. Conventional techniques for monitoring plant growth and health status rely on image processing and analysis, such as hyperspectral imaging, satellite imaging, infrared sensors, and Raman spectroscopy [12]. While effective, these systems still have several limitations, including the high cost of measurement instrumentation, time-consuming processes, high labor costs, and difficulty deploying to a large number of plants. Flexible mechanical sensors have emerged as strong candidates for real-time plant health measurement to support cultivation and management [13]. Flexible devices can be mounted on plant surfaces and, in some cases, minimally integrated into plants to measure vital parameters, including elongation (for growth rates), sap flow rate (for transpiration monitoring), light intensity (photosynthesis), moisture, and various analytes (e.g., pesticides and pathogens) [14]. These parameters serve as feedback signals to improve plant growth and crop management practices, including optimization of vital factors such as soil quality, irrigation, and light exposure [15]. Among these sensors, mechanical sensors have been an active area of research, particularly for growth measurement and water flow monitoring. Several sensing mechanisms and manufacturing technologies have been proposed to enable sensitivity, measurement range, and device installation [16]. Combined with wireless communication technologies, primarily Bluetooth, flexible mechanical sensors can capture the physiological conditions of plants and transmit the data to display and recording equipment, such as smartphones, computers, or cloud platforms, enabling further analysis of plant growth and metabolism and enhancing crop cultivation processes.

Given the exciting opportunities in this emerging field, this perspective provides an overview of recent progress and the utilization of flexible mechanical sensors for smart agriculture. It is noteworthy that there are a number of review papers on plant wearable sensors, and readers are recommended to refer to these works for a comprehensive overview of various technologies for plant phenotyping, including physical (e.g., elongation), chemical (e.g., gas and moisture), and optical (e.g., UV and light intensity) sensors [12,13,14,17]. With an emphasis on mechanical sensors, this paper highlights sensing mechanisms and their advantages and disadvantages in monitoring mechanical parameters, particularly growth rates and snap flows. The paper presents several examples of material choices, fabrication methods, and integration techniques to realize stand-alone sensing systems. The perspective also discusses key challenges and potential directions for future research and development. 

## 2. Mechanical Sensing Technologies for Smart Agriculture

### 2.1. Sensors for Elongation Measurement

Plant growth is typically measured by using strain sensors, where the elongation of plants induces strain in the sensing elements, resulting in either an electrical output or a shift in optical wavelength that can be detected by an integrated circuit or an optical interrogator. Requirements for strain sensors include excellent mechanical flexibility and stretchability, allowing them to attach smoothly to the curvilinear surface of plants and adapt to significant elongation during long-term monitoring.

**Figure 1:**
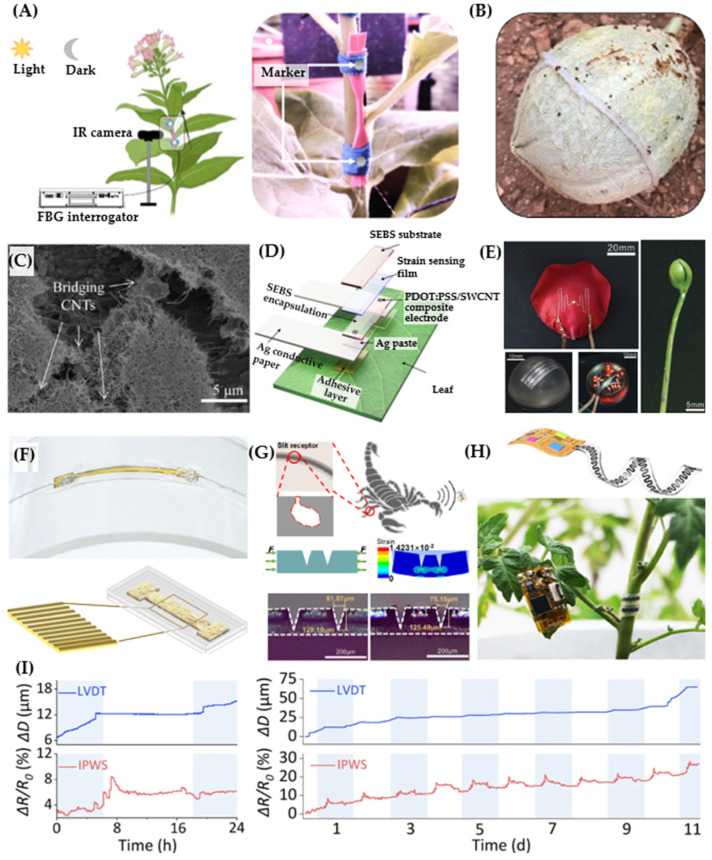
Strain sensors for growth monitoring. (**A**) FBG sensors in a dumbbell shape for elongation measurement [20]. (**B**) Ring–shaped FBG sensors for multidirectional strain measurement [20]. (**C**) Conductive ink using a composite of CNT and graphite flakes directly written on a soft substrate [23]. (**D**) Engineering PDOT: PSS for highly stretchable sensors [25]. (**E**) Hydroprinting of liquid metal on plant [27]. (**F**) Gold wrinkle structures to enhance the stretchability of strain sensors [29]. (**G**) Biomimetic strain sensors inspired by the slit receptor of scorpion. Bottom: Microscopic images of the double V-shaped groove with different distances. [33]. (**H**) Tendril–inspired structure for self-attaching pulse sensors. (**I**) Continuous stem diameter variation monitoring using the self-attaching sensor. (**Left**) Data measured on day 2. (**Right**) Data measured from 11 days of cultivation [34].

Some preliminary works have used off-the-shelf components, such as rotary encoders, to measure stem elongation. The key sensing principle of this method relies on converting plant expansion into rotational motion using a system of pulleys and wires [18]. This technique is relatively low-cost, simple, and effective for large plants, such as palms, which experience high elongation (e.g., several centimeters), where the entire device can be affixed to the plant body. However, due to its bulky size, this approach is not suitable for monitoring small and delicate plants, such as vegetables. A more efficient method for measuring smaller plants utilizes commercially available optical fibers. Optical fibers are widely used in various industrial systems, especially for monitoring strain in machines like ship hulls and airplanes, or infrastructures such as bridges and buildings. Presti et al. employed fiber Bragg gratings (FBGs) embedded in a silicone mixture (Dragon Skin™ 20, Smooth-On, Inc., Macungie, PA, USA) for flexible strain sensing [19]. The device has a dumbbell shape with overall dimensions of 48 mm × 8 mm × 1 mm, with a narrow section of 12 mm × 2 mm × 1 mm (Figure 1A). The use of highly stretchable silicone enhances the robustness of the FBG and improves its adherence to plants, simplifying the attachment process. As the plant elongates, it pulls on the flexible matrix, straining the encapsulated FBG, which leads to a shift in the Bragg wavelength (λ_B_). The sensor was tested to measure the elongation of tomato stems in both indoor and outdoor environments, with data collected using an FBG interrogator (FS22, HBM FiberSensing, Darmstadt, Germany) at a frequency of 1 Hz. Temperature compensation was applied to extract the sensor response to elongation. The data showed an increase in the Bragg wavelength of approximately 0.5 nm after 22 h of monitoring, corresponding to an elongation of 720 µm, demonstrating the high feasibility of fiber optic systems for growth measurement. Changing the dumbbell structure to a ring shape allowed for the measurement of fruit circumferential changes (Figure 1B) [20]. Attaching the ring-shaped FBG sensor to a watermelon revealed fruit expansion under various conditions. Specifically, the fruit showed dynamic circumferential changes, likely induced by both growth and waterlogging mechanisms. When exposed to direct sunlight, a considerable reduction in the Bragg wavelength was observed, which is attributed to fruit dehydration due to high temperatures, leading to a depression in water uptake. On the other hand, an increase in the Bragg wavelength was observed when the soil was watered, suggesting fruit expansion due to water uptake. The elongation of fruit and leaves can be measured in different orientations using biomimetic structures. By using six FBG structures arranged in a flower design, the same research group developed a wearable strain sensor that adapts to the curvilinear surfaces of fruits and leaves, enabling the measurement of growth in multiple directions [21]. The device was validated by measuring the dimensional changes in Cucumis melo and Nicotiana tabacum leaves under practical cultivation conditions, allowing for the study of anisotropic plant growth.

These examples demonstrate the potential of commercial FBG sensors and engineering techniques to improve their mechanical flexibility and compliance for plant growth monitoring. However, while FBG sensors are highly reliable and precise, their requirement for external FBG interrogators can make the system costly and bulky, posing a challenge for large-scale deployment. Additionally, the relatively large bending stiffness of FBG fibers and their limited stretchability make them less suitable for small, thin leaves and stems. Therefore, developing miniaturized sensors on flexible substrates is essential for monitoring the growth of small and delicate plants. Tang et al. developed a low-cost and rapid fabrication method for a flexible strain sensor using conductive ink made of chitosan mixed with graphite powders [22]. Chitosan is utilized due to its excellent biocompatibility, nontoxicity, and ability to bind the conductive graphite flakes together and, at the same time, to adhere the sensor to the substrate. A writing brush was used to apply the ink onto a soft substrate (Buna-N rubber), which was then left to dry for 15 min in air, resulting in a stretchable and flexible strain sensor. Various mixing ratios of chitosan and graphite were investigated, with the 1:2 ratio proving to be the most effective for writing on different substrates, including the surfaces of plants. This simple fabrication process offers a promising approach for on-site and disposable applications. The sensors were tested for strain sensing with strain (ε) up to 12%, showing a gauge factor (GF) of 2 (GF = [∆R/R]/ε). The sensor was then written directly onto fruits (e.g., cucumber and luffa) to capture their dynamic growth, showing an increase in resistance corresponding to an increase in the diameter of the fruit. Upon cutting the fruit from the plant, the resistance decreased significantly, indicating a reduction in metabolism in response to the lack of nutrients. Using this direct writing approach, the group further developed a carbon nanotube (CNT) and graphite mixture as a highly conductive ink to form sensors on plant surfaces (Figure 1C). Compared to the chitosan–graphite ink, this composite ink exhibited significantly enhanced stretchability due to the presence of CNT networks, which connect graphite flakes under large strains [23]. The multi-matrix composite ink allowed for large strains of up to 150%, with a high gauge factor of 48 at 50% strain, increasing to 352 at 150% strain. This large strain capacity is attributed to the significant increase in resistance caused by cracks forming between graphite flakes, which are bridged by CNT membranes. By connecting the strain sensors to a simple serial resistance circuit, an AD converter, and a microprocessor, data could be recorded and displayed on an LCD screen (LCD1602). The large measurement range allowed for the monitoring of fruit growth (*Solanum melongena* L.) over a 9-day period. The output from the sensors indicated a faster growth rate from midnight to 6 a.m., with a slower growth rate observed during the daytime. 

While direct ink writing sensors can effectively track fruit growth, this process typically occurs at a later stage. There is a need to develop sensors capable of measuring early growth stages, especially the vegetative stage, which is more sensitive and has greater demand for resources. Borode et al. addressed this need by developing highly stretchable strain sensors for monitoring early plant growth and optimizing water and fertilizer usage using polyaniline (PANI), one of the most common one-dimensional conductive polymers [24]. PANI nanoparticles were synthesized through chemical oxidative polymerization of aniline in HCl and then in situ dip-coated onto an elastic band during the polymerization process. Compared to the low-cost, rapid direct writing method, the dip-coating technique requires more complex fabrication resources and additional steps for polymerization. However, it is an industrially standardized process (e.g., used in rubber glove manufacturing) and highly suitable for scalable production. The PANI sensors were characterized over a strain range of 0 to 100%, showing two linear regimes: from 0 to 50% with a gauge factor of 3.8, and from 50% to 100% with a gauge factor of 2.5, comparable to that of direct ink writing graphite sensors. The sensor was then attached to the stems of sunflowers and soybeans for real-time measurement of their growth over a two-day period. Based on the relative resistance change observed on day 1 (∆R/R = 60%), rapid growth in sunflowers was detected during the dark cycle, while the light cycle showed a more stable growth rate. In contrast, soybeans exhibited a linear growth pattern during both dark (8 h) and light (16 h) cycles. The measured growth factors depended on the plant stage, species, and location on the stem, highlighting the importance of strain sensors for optimizing and managing cultivation.

The work reported by Tang et al. and Borode et al. demonstrates the effectiveness of strain sensors for monitoring stem elongation and fruit expansion [23,24]. However, the measured strain was still relatively low (less than 200%), considering the significant increase in stem length over a long period of growth. To further extend the measurement range, Wang et al. developed a conjugated polymer-based strain sensor with an operating strain of over 400% (Figure 1D) [25]. The sensor is ultra-lightweight (45 mg), environmentally stable with a degradation rate of 0.0008/h, and highly reproducible with a coefficient of variation of 14.4%. To achieve these features, the authors employed a conductive polymer material system based on PEDOT, a material widely used in biomedical applications due to its flexibility and global availability. It should be noted that the intrinsic stretchability of PEDOT is only around 10%, which is insufficient for long-term plant growth measurement. This limitation was overcome by adding fluorinated surfactant Zonyl and ionic additives to weaken the Coulomb interaction between PEDOT and PSS, enhancing the wetting of the substrate. These modifications increased the PEDOT domain sizes, providing a high conductivity of 153.5 S/cm and extending the measurement range to 700% by delaying crack onset and altering the crack morphology. The conductive PEDOT was coated on and encapsulated in a highly stretchable substrate of styrene–ethylene–butylene–styrene (SEBS), which has a large fracture strain of 800%. The strain sensor was used to monitor the growth of Avena sativa (oats), where one side of the sensor was fixed to the sheath and the other to the blade. The sensor was able to measure the elongation of Avena sativa, which increased from 8 mm to 28 mm, corresponding to a high strain of 400%. The sensor effectively captured different growth patterns when the plant was subjected to electrical stimulation (e.g., switching the lighting on and off every 3 h). The sensor also featured remote sensing functionality by integrating it with a SNAP wireless module based on the IEEE 802.15.4 standard [26], demonstrating data transmission to a PC over a distance of at least 30 m. These features provide a powerful tool for investigating fundamental plant biological mechanisms by enabling precise and remote tracking of plant growth.

The direct ink writing and dipping methods allow for the rapid, low-cost fabrication of soft strain sensors, with fabrication times on the order of minutes. However, these techniques still require an elastic substrate to host the conductive sensors and attach them to different parts of plants. Due to the rapid growth of plants, which alters their morphology, wearable strain sensors must be inherently stretchable and adaptable to these morphological changes. In addition, for small plants, monitoring growth from seeding requires ultrathin sensing elements and substrates that can morph with the complex structural changes during growth. Compared to the direct ink writing method, hydroprinting (also known as water printing) offers unique features for creating ultrathin and lightweight 2D structures in water, which can then be transferred onto plants without carrier films. Jiang et al. developed morphing circuits and sensors for plant monitoring using gallium-based liquid alloy (LA) on a water-soluble polyvinyl alcohol (PVA) film (Figure 1E) [27]. The prepared seedling is aligned with the LA structures and immersed in water, allowing the LA circuit on the PVA gel to transfer and adhere to the plant epidermis. This attachment occurs due to surface tension and hydrostatic pressure during the immersion process. After removing the seedling from the water and clearing any residual PVA gel, the LA circuits and sensors remain seamlessly attached to the plant surface, ready for growth monitoring. As the resistance of liquid metal changes when subjected to mechanical elongation, this phenomenon was utilized to monitor the length increase in bean sprouts over several days. Thanks to the mechanical compliance of the liquid metal, the heights of pristine and hydroprinted bean sprouts were comparable, indicating that coating the sprout with a thin layer of LA sensor did not affect the plant development. As the bean sprouts grow from the bean-shaped cotyledons, the hypocotyl exhibits dynamic development, particularly near the cotyledons, while the lower section near the radicle remains almost unchanged. The mechanical flexibility and stretchability of LA help retain a conformal attachment between the strain sensor and the epidermis of the sprout. The elongation of the sprout leads to an increase in the length of the LA strain sensor and a reduction in its cross-sectional area. The relative resistance has a quadratic relationship with the relative length, enabling dynamic measurement of plant development.

The above examples highlight the advantages and progress in the development of conductive materials for growth monitoring. It is noteworthy that the majority of commercially available mechanical sensors are still built on conventional inorganic materials, such as metallic or silicon strain sensors, where scalable processes such as thin film deposition, wet/dry etching, and laser machining can be utilized for mass manufacturing [28]. Considering the key advantages of these materials, several engineering approaches have been proposed to tailor the low cost and availability of metallic materials for highly flexible and stretchable strain sensors for smart agriculture. One example is the work by Nassar et al., using a nanothin film of Au deposited on a micro-thick PDMS substrate for a lightweight, highly stretchable strain sensor (Figure 1F) [29]. The intrinsic tensile strain of Au is limited to a few percent, which is not sufficient for continuous, long-term plant growth measurement. The authors applied a pre-straining technique to the elastomer (PDMS) during Au deposition, creating a wrinkle structure. This out-of-plane wavy structure mitigates the strain induced in the Au film, enhancing the stretchability up to 35%. The sensor demonstrated good linearity within a strain range of up to 22% and a gauge factor of 3.9, comparable to typical metal strain gauges. The sensor was connected to a Bluetooth module (PSoc) and a rechargeable battery, allowing for remote sensing applications. The device was attached to the stem of a barley plant grown indoors and lucky bamboo shoots for a period of two days. The authors suggested different installation methods and locations for each plant, depending on their geometry and growth behaviors. The data demonstrated the efficiency of the wavy Au strain sensors, which captured the growth of stems in both the barley plant and lucky bamboo, with growth rates of 2.7 cm/day and 905 µm/day, respectively. 

In addition to the pre-strain technique, smart structures like serpentine designs or cut patterns can enhance measurement range and sensitivity. Zhao et al. utilized serpentine structures in copper (Cu) interconnects to enhance the stretchability of strain sensors up to 150% in the y-direction and 60% in the x-direction [30]. Interconnecting the 2D highly stretchable Cu trace with a CNT sensing element enabled the monitoring of growth in a Scindapsus aureus leaf over two days. Distributing two sensing elements within the serpentine network allowed for the measurement of leaf expansion in different directions, indicating faster growth in the y-direction compared to the x-direction. The growth rate during the nighttime was found to be more significant than during the daytime. To further enhance the mechanical stretchability of sensors and circuits, 2D structures can be engineered into 3D configurations using various techniques such as pre-strain, kirigami, and origami. This new class of unconventional electronics has been widely employed in healthcare applications, where 3D configurations adapt seamlessly to the highly deformable nature of human skin [31]. Building on this concept, Zhang and co-workers pioneered the use of 3D structures for wearable agricultural sensors [32]. The authors selectively thinned a 100-micrometer-thick polyimide (PI) film using laser engraving, creating arch-shaped creases. These creases, with tunable mechanical properties determined by their geometrical dimensions, exhibit much lower bending stiffness compared to other areas of the PI film. This allows the 2D precursors to buckle when their two ends are fixed, forming origami-like structures. The laser engraving process was further utilized to produce laser-induced graphene (LIG) from the PI film, enabling seamless integration of functional materials onto the 3D mesostructures. Positioning the LIG at strain-concentrated regions of the out-of-plane origami configurations allowed the measurement of leaf elongation with strain levels of up to 100%, as demonstrated in pumpkin leaves. In addition to excellent stretchability, the device also maintains limited surface contact with the plant, minimizing interference and hindrance to plant growth. In another work, inspired by the slit receptor of a scorpion, Huang et al. developed a highly sensitive wearable plant sensor using a nanometer-thick silver (Ag) film on a polyethylene glycol terephthalate (PGT) substrate (Figure 1G) [33]. The biomimetic structure, inspired by the silt sensillum of a scorpion (which can be stimulated by weak stimuli, such as an insect walking), was created through V-shaped groove patterns on the PGT. The sensor operates based on the tunneling current effect. Specifically, when a strain is applied to the Ag-coated PGT sensor, the distance between the Ag conducting paths at the V grooves changes, leading to a change in the tunneling current. The sensor was characterized for strains up to 1%, showing high gauge factors ranging from 49 to 182. While the measurement range is relatively small, its large gauge factor allows for the detection of small changes in plant circumference, as demonstrated in a lucky bamboo plant. A biomimetic approach was also employed to facilitate sensor installation on plant surfaces. Most stretchable strain sensors require an adhesive layer to attach to the plant, which presents limitations such as adhesion strength changes due to environmental factors. To overcome this issue, Zhang et al. developed an adaptive winding strain (AWS) sensor inspired by the spiral shape of plant tendrils (Figure 1H) [34]. The AWS device consists of three layers, with laser-induced graphene (LIG) sandwiched between two transparent Ecoflex layers. The LIG sensing layer was formed by laser engraving a thin film of PDMS coated with phenolic resin, then mechanically transferred to an Ecoflex layer using the low surface energy of PDMS. The LIG on Ecoflex film was bonded to another pre-strained Ecoflex layer. Releasing the pre-strained Ecoflex resulted in a tendril structure, generated by the mismatch between the strain levels in the top and bottom Ecoflex layers. The sensor was connected to a wireless module (WIFI wireless data transmission) developed on a flexible PCB circuit. The tendril configuration enabled seamless integration of the sensor with the stem of a tomato plant without using any adhesive layer or tape. The sensor continuously monitored the growth of the tomato stem for 11 days, showing that when the transpiration rate exceeded the root water absorption rate, the stem shrank, which was detected by a change in LIG resistance. Figure 1I plots the stem diameter variation (SDV) during 11 days of cultivation. The expansion of the stem diameter measured using the AWS device is consistent with that recorded using a commercial linear variable transducer. The data recoded at day 2 (Figure 1I, left) show that the SDV mainly occurred during nighttime and was relatively stable during daytime. A similar trend was observed for other measurement days, in which the stable SDV during daytime is attributed to an equality between the transpiration rate of plants and the water absorption rate of roots. The dynamic detection of stem expansion and shrinkage (plant pulses) under varied soil moisture conditions demonstrated the capability of AWS in assessing the water status of plants.

### 2.2. Sap Flow Monitoring Technologies

**Figure 2:**
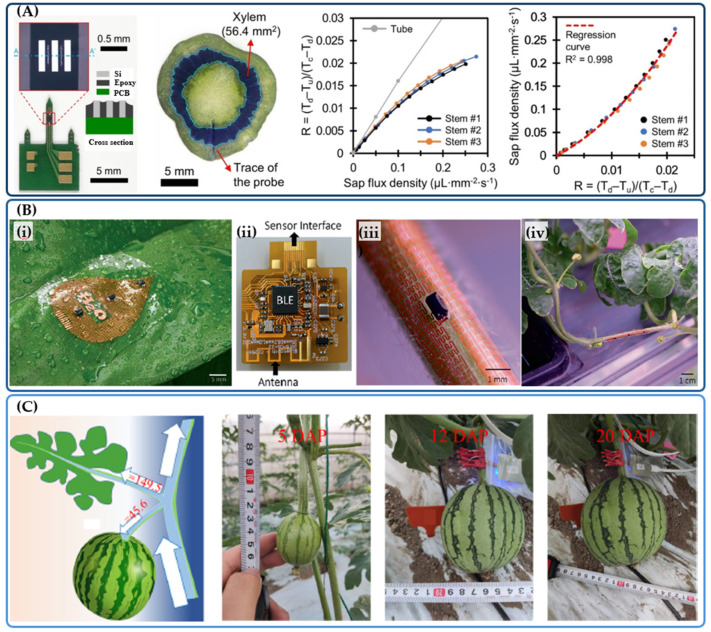
Miniaturized sap flow sensors. (**A**) Minimally invasive flow sensor PCB needle [41]. Left: Photograph of a needle with a heater and two temperature sensors. Middle: An image showing the penetration of the needle into the stem. Right: Calibration curves showing the relationship between sap flow and the resistance change. (**B**) Flexible flow sensors fabricated using flexible printed circuits [44]. (**i**) The sensor contains one heater located at the center and two temperature sensors on each side. (**ii**) Integration with a BLE circuit for wireless sensing. (**iii**) Installation of sensors on stem. (**iv**) An image of a flow sensors with two temperature sensors on a watermelon stem. (**C**) Sensor attached to different branches for long–term flow measurement during three growth stages of watermelon. DAP: days after pollination [45].

The measurement of sap flow is critically important for understanding plant physiology and plant responses to environmental variations such as temperature, sunlight, and soil water content [35]. While sap flow sensors are commercially available and actively used to monitor water absorption and metabolism, they are relatively bulky and only suitable for large, rigid plants [36]. Their large needles make them unsuitable for measuring flow in soft and small plants. To address these limitations in existing sap flow sensors, Bae et al. developed MEMS microneedle thermal probes that can be implanted into stems to measure sap flow through the xylem [37]. The thermal probe was fabricated from a 300 μm Si wafer with a sharp tip (less than 50 μm in width) to facilitate sensor penetration into the stem. The sensor operates based on the hot-wire anemometry concept, where constant electric power applied to an Au heater increases its temperature via the Joule heating effect [38,39]. When there is sap flow in the stem, the enhanced heat convection caused by the liquid flow reduces the temperature difference between the heated and unheated probe. By measuring the change in this temperature difference (Δ*T*), sap flow can be estimated using King’s law for hot-wire anemometry: $u=a\cdot {\left[(∆T_{M}-∆T)/∆T\right]}^{b}$, where u is the sap flux density and ∆*T* is the temperature difference when there is no sap flow. The total sap flow is calculated from the sap flux: *F* = *u* · *Sa*, where *Sa* is the cross area of the stem. Since heat transfer in the xylem involves both conduction and convection within the vascular bundles, the sensor was further calibrated using a plant stem sample with water supplied from an Erlenmeyer flask at flow rates ranging from 0 mm/s to 3 mm/s [37]. Calibration showed that heat conduction through the vascular bundle network leads to a more significant temperature drop in the xylem compared to typical silicone channels used for calibration. After calibration, the microneedle sap flow sensor was tested on a tomato plant grown in a greenhouse for 36 days. The small size of the probe caused minimal effect on the plant, with the flow rate being measurable from the second day of implantation, once plant tissues had formed around the penetration site. Experimental results showed that the sap flow rate correlates with the daily cycle of the tomato plant in response to variations in air temperature and solar radiation. On sunny days, sap flow quickly increased after sunrise due to the rise in solar radiation and air temperature, peaking at noon and then decreasing after sunset as the air temperature dropped. The small footprint of the microneedle sensors, combined with the miniaturized sensing element (heater), suggests high potential for multimodal sensing in plant health monitoring. Using the same single-probe sensing concept, Ino et al. developed a sap flow sensor incorporating a resistance temperature detector (RTD) and a heating filament [40]. The sensor was used to monitor sap flow in *Solanum lycopersicum* L. grown in a culture medium to investigate the influence of light intensity and vapor pressure deficit. The results showed a clear correlation between increased sap flow velocity and higher light intensity and vapor pressure deficit, consistent with the diurnal variations of sap flow observed in the stems of stressed plants and shrubs. A recent work reported by Kim and Lee demonstrates the use of printed circuit boards (FCB) to replace silicon for the development of microneedles (Figure 2A) [41]. Different from Si, which exhibits mechanical brittleness, the use of PCB can reduce device damage when inserting into plant stems. In addition, using two temperature sensors on each side of the heater on the PCB enables the measurement of flow direction. Another benefit of microneedle platforms is their capability to extract biofluid from plants for further electrochemical analysis. Integrating electrodes on microneedle, therefore, serves as a multimodal device that can detect not only snap flow but also other signaling molecules such as glucose concentrations and pH levels [42,43]. 

Microneedles, while reducing the invasiveness to plants compared to conventional millimiter-scale sap flow sensors, still represent a certain level of tissue or xylem damage. The development of wearable sensors that can monitor sap flow without penetrating the xylem is of particular interest to avoid causing plant damage and stress. Chai et al. developed the first flexible flow sensor that can be attached to the surface of the stem to track sap flow inside (Figure 2B) [44]. The sensors were created using a common technique for manufacturing flexible printed circuits (FPC), where copper on polyimide films was laser-cut into highly stretchable serpentine structures to provide mechanical flexibility. Two commercial positive temperature coefficient (PTC) thermistors placed on either side of a heater form an anemometric-type flow sensor. The cut-out structure of the copper trace allows light to pass through, thus maintaining the photosynthesis process. Applying a constant power to the heater raises the temperature of the two sensors equally in a flow-free state. When sap flow occurs, the forced heat convection from the liquid flow causes an asymmetry in the output temperature. Measuring the temperature difference between the two PTC thermistors determines the flow direction and velocity. Connecting the sensor to a BLE module developed on a flexible FCB circuit allows data to be sent to a smartphone app for remote control and monitoring. The sensors were then attached to the basal stems and two adjacent branches near a watermelon fruit to monitor water allocation. Data recorded from these sensors showed that most of the water from the basal stem was allocated to the leaf branch during the daytime, due to photosynthesis. At nighttime, however, the flow to the leaf branch stopped, and water primarily flowed to the branch connected to the watermelon fruit. These observations indicate that fruit fresh weight accumulation mainly occurs at night, contradicting the conventional hypothesis that fruit growth occurs during photosynthesis. These interesting findings highlight the promising potential of wearable flow sensors for plant phenotyping.

Utilizing this advanced flexible sensing platform, the same research group further investigated watermelon fruit growth over a long period (35 days) across three different stages (7–20 days, 20–29 days, and 29–35 days) (Figure 2C) [45]. Data from the soft anemometric sap flow sensors showed that water allocation depends on the growth stage. Specifically, during the first stage, sap flow into the fruit gradually halts after sunrise due to increased leaf transpiration, followed by a sharp rise in flow after noon that continues until the next morning, coinciding with fruit expansion. In the second stage, there is a significantly shorter sap inflow period from noon to night and increased outflow from the fruit, aligning with enhanced leaf transpiration after sunrise, reflecting slower fruit growth during this phase. In stage 3, the sap flow follows a diurnal cycle, but the sap inflow into the fruit at nighttime is nearly balanced by outflow during the day, leading to a stable fruit phenotype. Long-term sap flow measurements could provide valuable information for yield prediction and improved cultivation.

## 3. Summary and Outlook

Various types of mechanical sensors have been developed and demonstrated for plant growth and sap flow monitoring. While FBG sensors offer high sensitivity, their requirement for optical instruments for signal readout represents a significant burden for large-scale deployment. The low maximum strain tolerance of fiber compared to polymer materials limits their application for long-term monitoring. To improve mechanical compliance, soft, transparent materials can be considered to develop highly stretchable FBG (e.g., using PLGA—poly lactic-co-glycolic acid) sensors [46]. Conductive ink directly deposited onto or mixed with polymers offers excellent stretchability, in some cases with strain capabilities of up to 500%, suitable for long-term monitoring. A limitation of composite materials is their electrical and mechanical stability, which can be affected by temperature fluctuations and moisture absorption. Encapsulation and coating strategies are needed to enhance the long-term stability of these sensors for practical field applications. Inorganic materials such as gold (Au) and silicon (Si) thin films, together with FPC (copper on polyimide), are the mainstream materials for epidermal sensors and electronics in healthcare applications. The use of 3D configurations formed by buckling techniques can be considered to improve the measurement range of inorganic material-based sensors [32,47]. Three-dimensional configurations have been demonstrated for multimodal sensing; however, they are prone to signal crosstalk, where mechanical sensors not only respond to strain but are also sensitive to temperature. Some strategies recently developed for healthcare applications can be utilized to minimize the cross effect. In particular, placing metal traces symmetrically above and below the neural plane of a 3D polyimide structure can create a tensile and compressive sensing element that compensates for thermal effects using a Wheatstone bridge [31]. 

Flow sensors developed using anemometric configurations have been demonstrated for sap flow measurement. Microneedle sensors offer higher sensitivity, but their invasiveness can cause plant tissue injury and stress. Reducing the needle size to minimize tissue damage can lead to localized flow measurements, which may not accurately represent the total sap flow rate in the stem. Further experimental studies and modeling are needed to reduce the uncertainty in invasive flow measurements. Wearable sensors are emerging as promising candidates for flow measurement. However, the relative position between the xylem and the sensing element (temperature and heater) is likely to change. Therefore, precisely measuring the sap flow rate based on lab-calibrated data can be challenging. In this regard, utilization of an array of wearable anemometers wrapping around stems can be considered a potential solution for reliable snap flow monitoring. In addition, environmental factors such as moisture, and the fluctuation of the surrounding temperature can affect the forced heat convection, causing error in flow measurement. Embedding an anemometer in flexible, thermal insulation structures may help mitigate these external influences.

Aside from the sensing concept and device configuration, energy supply for agriculture is another critical concern. Self-energy harvesting systems, utilizing solar energy or moisture, are potential solutions for powering a highly distributed network of agricultural sensors. In addition to energy harvesting, reducing device footprints is critically important to avoid undesirable growth interference. In particular, in a recent systematic study reported by Xiao et al., mechanical pressure, hindrance of gas exchange, hindrance of light acquisition, and mechanical constraint can adversely affect leaf growth [48]. For instance, experiments performed on Peperomia tetraphylla indicate a significant decrease in leaf growth rate when increasing the covering surface area of wearable sensors from 10% of the leaf surface area (corresponding to the maximum length growth of 3.3 mm after 17 days) to 100% (corresponding to the maximum length growth of 2 mm after 17 days). A decrease in the growth rate was also observed in the same type of plant when increasing mechanical pressure (device mass). Specifically, the maximum length of growth reduced from 3.3 mm (with a 0.3 mg wearable device) to 1.3 mm (with a 1.5 mg device). Therefore, device mass, dimensions, and porosity need to be taken into account when designing plant wearable sensors. 

The development of mechanical sensors for agriculture is an emerging, non-traditional research area. However, this new class of devices has demonstrated their potential for continuous plant growth monitoring, showing trends that correlate with environmental parameters. Further research and development of soft, wearable, and implantable sensors, including material and structural design, energy solutions, wireless signal transmission, and data analysis (e.g., using machine learning), can open exciting opportunities in this field and bring them closer to practical smart agriculture systems.
